# Silencing of IFN-stimulated gene transcription is regulated by histone H1 and its chaperone TAF-I

**DOI:** 10.1093/nar/gku485

**Published:** 2014-06-14

**Authors:** Shinichi Kadota, Kyosuke Nagata

**Affiliations:** Department of Infection Biology, Faculty of Medicine, University of Tsukuba, 1-1-1 Tennodai, Tsukuba 305-8575, Japan

## Abstract

Chromatin structure and its alteration play critical roles in the regulation of transcription. However, the transcriptional silencing mechanism with regard to the chromatin structure at an unstimulated state of the interferon (IFN)-stimulated gene (ISG) remains unclear. Here we investigated the role of template activating factor-I (TAF-I, also known as SET) in ISG transcription. Knockdown (KD) of TAF-I increased ISG transcript and simultaneously reduced the histone H1 level on the *ISG* promoters during the early stages of transcription after IFN stimulation from the unstimulated state. The transcription factor levels on the *ISG* promoters were increased in TAF-I KD cells only during the early stages of transcription. Furthermore, histone H1 KD also increased ISG transcript. TAF-I and histone H1 double KD did not show the additive effect in ISG transcription, suggesting that TAF-I and histone H1 may act on the same regulatory pathway to control ISG transcription. In addition, TAF-I KD and histone H1 KD affected the chromatin structure near the *ISG* promoters. On the basis of these findings, we propose that TAF-I and its target histone H1 are key regulators of the chromatin structure at the *ISG* promoter to maintain the silent state of ISG transcription.

## INTRODUCTION

Transcriptional regulation is accomplished mainly by the regulatory elements such as promoters and enhancers, those of which have a variety of binding sites for sequence-specific transcription factors, and specify characteristic chromatin structures mediated by nucleosome positioning, specific histone modifications, histone variants and other factors (1,2). For the transcription of type-I interferon (IFN)-stimulated genes (ISGs), *ISG* promoters containing sequence motifs, known as IFN-stimulated response element (ISRE), are the binding sites of the sequence-specific transcription factors activated by IFN stimulation. The chromatin structure and histone modification around ISRE are regulated by coactivators for ISG transcription (3,4).

IFN, in particular type-I IFN, plays a critical role in cellular antiviral mechanisms by inducing immediate transcription of ISGs, which encode proteins that not only have anti-viral activities but also affect host-cellular events, such as cell death, messenger ribonucleic acid (mRNA) degradation and translational arrest, through the IFN signaling pathway, called the JAK-STAT pathway (5). In the case of stimulation by type-I IFN, the IFN-stimulated gene factor 3 (ISGF3) complex consisting of transcription factors, signal transducers and activators of transcription (STAT)1, STAT2 and IFN regulatory factor 9 (IRF9) bind to ISREs in the *ISG* promoters, facilitating transcription initiation complex formation and thereby promoting the transcription of ISGs (3,5). In addition, ISG transcription is regulated by several different kinds of coactivators (4). STATs interact with histone acetyltransferases (HATs), including p300/CBP and GCN5, and GCN5 acetylates histones on the *ISG* promoter in an IFN-dependent manner (6,7). Interestingly, inhibition of histone deacetylase (HDAC) activity, which opposes activity of HAT, leads to a global impairment of the ISG transcription (8–10). In addition, pp32, a major component of the inhibitor of acetyltransferase (INHAT) complex (11), is involved in the maximal induction of ISG transcription (12). BRG1, an adenosine triphosphate (ATP)-dependent nucleosome remodeling factor and a subunit of the SWI/SNF complex, interacts with STAT2 in response to IFN, facilitates the chromatin remodeling of the *ISG* promoter region and promotes ISG transcription (13–15). BAF200, a subunit of the SWI/SNF complex, has been found to be required for selective ISG transcription (16). These studies suggest that ISG transcription via *ISG* promoters is under the control of the combined effects of histone modification and specific chromatin structures. As proteins encoded by *ISGs* not only have anti-viral activities but also affect host-cellular events (5), the ISG transcription needs to be silenced in the IFN-unstimulated condition. However, it is unclear as to how the chromatin structure of *ISG* promoters is regulated to be in the unstimulated state, namely the transcriptionally silent state, in the absence of IFN, and what kind of factors maintain the transcriptionally silent state of the *ISG* promoter remains unknown.

Linker histone H1 binds near to the entry and exit sites of the nucleosome core particle (NPC), which consists of a 147-base-pair (bp)-long deoxyribonucleic acid (DNA), wrapped around a histone octamer consisting of two copies each of the core histone proteins H2A, H2B, H3 and H4, and facilitates the higher order chromatin structure (17–19). Histone H1-dependent chromatin dynamics have been shown to be important in a variety of biological phenomena and in the transcriptional regulation of a certain gene (20–24). In addition, fluorescence recovery after photobleaching analyses demonstrated that histone H1 is a highly mobile chromatin component compared with the core histones and that the binding of histone H1 onto the nucleosome is transient (25,26). These findings suggest that histone H1 continuously associates with and dissociates from the chromatin, but it is unclear as to what kind of mechanisms and factors are involved in regulating histone H1 dynamics and H1-dependent chromatin structural alterations.

Several factors are involved in the regulation of histone H1 dynamics. High mobility group proteins weaken the binding of histone H1 to the nucleosome by dynamically competing for chromatin binding sites (27). Histone variant H3.3, which is incorporated into nucleosomal chromatin in association with active gene expression, counteracts the association of histone H1 (28). In addition to these proteins, template activating factor-I (TAF-I, also known as SET) regulates histone H1 dynamics as a histone chaperone (29). TAF-I was first identified as a stimulator of adenovirus DNA replication *in vitro*, and subsequent studies revealed that TAF-I acts as a histone chaperone for histones H3-H4 and linker histone H1 (29–33). TAF-I is associated with histone H1 in mammalian somatic nuclei and shows histone H1 chaperone activity *in vitro* (29).

In this study, we identified a novel mechanism for the silencing of ISG transcription by histone H1 and TAF-I. TAF-I knockdown (KD) facilitates ISG transcription during the early stages of transcription following IFN-mediated induction from the transcriptionally silent state. In TAF-I KD cells, the transcription factor levels on the *ISG* promoter were increased during the early stages of transcription following IFN induction, and the amount of histone H1 on the *ISG* promoter was significantly decreased from the transcriptionally silent state to the early stages of transcription following IFN induction. The domain of TAF-I that is essential for the histone H1 chaperone activity was also required for the TAF-I functions in ISG transcription. Histone H1 KD increased ISG transcription, as did TAF-I KD. Double TAF-I and histone H1 KD did not additively affect ISG transcription. The micrococcal nuclease (MNase) sensitivity of the *ISG* promoter was increased in both TAF-I KD and histone H1 KD. Taking these findings together, we propose that TAF-I recruits histone H1 to the *ISG* promoter through its histone H1 chaperone activity, suppresses transcription complex formation on the *ISG* promoter and restricts aberrant transcription under the transcriptionally silent state.

## MATERIALS AND METHODS

### Cell culture and antibodies

HeLa S3 and HEK293 cells were grown in Dulbecco's modified essential medium supplemented with 10% fetal calf serum. Antibodies used in this study are as follows: Anti-STAT1α/β (sc-346; Santa Cruz Biotechnology), anti-STAT2 (sc-476; Santa Cruz Biotechnology), anti-IRF9 (sc-10793; Santa Cruz Biotechnology), anti-phospho-(Tyr701) STAT1 (#9171; Cell Signaling Technology), anti-phospho-(Tyr689) STAT2 (#07-224; Upstate Biotechnology), anti-phospho-(Ser727) STAT1 (#06-802; Upstate Biotechnology), anti-Pol II (sc-899; Santa Cruz Biotechnology), anti-β-Actin (A5441; SIGMA), anti-Histone H3 (ab1791; Abcam), anti-acetyl-Histone H3 (06-599; Millipore), anti-Histone H1.2 (ab4086; Abcam), anti-Flag (F3165; SIGMA) and anti-TAF-Iα/β (monoclonal antibody KM1725) (34) antibodies.

### Preparation of plasmids

To construct plasmids expressing short interfering RNA (siRNA)-resistant Flag-TAF-Iα, Flag-TAF-IαΔC, Flag-TAF-Iβ and Flag-TAF-IβΔC, DNA fragments were amplified by polymerase chain reaction (PCR) using plasmids expressing His-TAF-Iα and His-TAF-Iβ (29) as templates and the following primer sets: 5′-GCGGATCCATGGCCCCTAAACGCCAGTC-3′ and 5′-ATGTGCTCTATTGCCTCCTGTTGTTCTTTTTCTCCCTTCTTCG-3′ for the N-terminal region of TAF-Iα (Nα), 5′-GCGGATCCATGTCGGCGCAGGCGGCCAA-3′ and 5′-ATGTGCTCTATTGCCTCCTGTTGTTCTTTTTCTGAGGTCTCGTC-3′ for the N-terminal region of TAF-Iβ (Nβ) and 5′-ACAGGAGGCAATAGAGCACATTGATGAAGTACAAAATGAAATAG-3′ and 5′-GCGAATTCTTAGTCATCTTCTCCTTCAT-3′ for the C-terminal region of TAF-Iα/β (Cα/β). To generate full-sized TAF-Iα/β and TAF-Iα/βΔC complementary DNA (cDNA), PCR was performed using Nα or Nβ and Cα/β as templates and the following primer sets: 5′-GCGGATCCATGGCCCCTAAACGCCAGTC-3′ and either 5′-GCGAATTCTTAGTCATCTTCTCCTTCAT-3′ for full-sized TAF-Iα or 5′-GCGAATTCTTACATATCGGGAACCAAGTAGT-3′ for TAF-IαΔC, and 5′-GCGGATCCATGTCGGCGCAGGCGGCCAA-3′ and either 5′-GCGAATTCTTAGTCATCTTCTCCTTCAT-3′ for full-sized TAF-Iβ or 5′-GCGAATTCTTACATATCGGGAACCAAGTAGT-3′ for TAF-IβΔC. The amplified cDNA fragments were digested with BamHI and EcoRI and cloned into pcDNA3.1(+)-Flag vector that had been digested with same enzymes. pU6-puro-siTAF-I and pU6-puro-siEGFP plasmids, which express 21-nucleotide-long hairpin-type siRNAs against TAF-I and EGFP, respectively, under the control of the U6 promoter were described previously (35,36). To generate pU6-puro-siH1 plasmid, which expresses 21-nucleotide-long hairpin-type siRNA against histone H1.2, 5′-terminal oligonucleotides, 5′-CACCGCTTCTTTAGACTTAACAGGAGTGTGCTGTCCTCTTGTTGAGTTTAAAGGAGCTTTTT-3′ and 5′-GCATAAAAAGCTCCTTTAAACTCAACAAGAGGACAGCACACTCCTGTTAAGTCTAAAGAAGC-3′ were phosphorylated, annealed each other and cloned into pU6-puro (36) that had been digested with BspMI.

### siRNA-mediated KD and western blot analysis

Cells were transfected with pU6-puro-siTAF-I, pU6-puro-siH1 or pU6-puro-siEGFP using Gene Juice (Novagen) according to the manufacturer's protocol. After 16 h post transfection, puromycin was added in the medium at the concentration of 2 μg/ml and cells were further incubated for 20–24 h. After selection in the presence of puromycin, cells were transferred to the medium in the absence of puromycin and further incubated for 48 h. siRNAs against TAF-I and histone H1.2 were commercially purchased (Stealth siRNA; Invitrogen). siRNAs were introduced into HeLa S3 cells with Lipofectamine RNAiMAX (Invitrogen) according to the manufacturer's protocol. Negative control Stealth siRNA (12935-200; Invitrogen) was used as a negative control. Cells were lysed in a cell lysis buffer [10-mM Tris-HCl, pH 7.9, 300-mM NaCl, 1-mM ethylenediaminetetraacetic acid (EDTA), 1% NP-40, 10% glycerol, 5-mM β-D-glycerophosphate, 1-mM sodium fluoride, 1-mM sodium orthovanadate and 1-mM phenylmethylsulfonyl fluoride (PMSF)] and incubated at 4°C for 30 min with constant rotation. After centrifugation, the supernatant fraction was separated on 7.5–12.5% sodium dodecyl sulphate-polyacrylamide gel electrophoresis (SDS-PAGE) and electroblotted to a nitrocellulose membrane. Nitrocellulose membranes were incubated with specific antibodies, followed by incubation with horseradish peroxidase-conjugated goat anti-rabbit antibodies (Amersham), biotinylated anti-rabbit Ig (Amersham) or biotinylated anti-mouse Ig (Amersham). Streptavidin-alkaline phosphatase (Amersham) or streptavidin-horseradish peroxidase (Amersham) was used for detection of biotinylated Igs.

### Total RNA extraction and quantitative real-time PCR

Total RNA was prepared from cells treated with or without IFN-β (1000 IU/ml; Toray) using RNeasy minikit (Qiagen) and RNase-free DNase I (Qiagen), and subjected to reverse transcription using SuperScript III Reverse Transcriptase (Invitrogen) with oligo-dT primer. Synthesized cDNA was used for quantitative real-time PCR (qRT-PCR) using FastStart SYBR Green Master (Roche) with specific primer sets, 5′-CAGATCACCCAGAAGATCG-3′ and 5′-CCCTTGTTATTCCTCACCAG-3′ for *ISG15* mRNA, 5′-ACACCTGAAAGGCCAGAATGAGGA-3′ and 5′-TGCCAGTCTGCCCATGTGGTAATA-3′ for *ISG56* mRNA, 5′-GTTACTGGTATTCGGCTCTG-3′ and 5′-GGTGTGTGGGTATAAACTGC-3′ for *IFITM1* mRNA, 5′-GACACGGTTAAAGTGTGGAG-3′ and 5′-GGTACTGGTTGTCAGGATTC-3′ for *ISG54* mRNA, 5′-AAACCAAGGCACAGTGGAAC-3′ and 5′-TAGCAAAATTGAGGCCAAGG-3′ for *IL8* mRNA, 5′-CTCCGAGACTTTCGAGGAAATAC-3′ and 5′-GCCATTGTAGTTGGTAGCCTTCA-3′ for *IκBα* mRNA, 5′-AGCCAAAAGGGTCATCATCTC-3′ and 5′-GGACTGTGGTCATGAGTCCTTC-3′ for *GAPDH* mRNA and 5′-AGCCAGTGCCTTGTGTGT-3′ and 5′-CAGCTCTGACACCGACAT-3′ for *ISG15* pre-mRNA.

### Chromatin immunoprecipitation assay

Cells were treated with or without IFN-β (1000 IU/ml) or trichostatin A (TSA, 0.1 or 1 μM; SIGMA) for indicated periods followed by fixation with 1% formaldehyde at room temperature for 15 min. Fixation was quenched by the addition of glycine at the final concentration of 125 mM, and cells were washed twice with phosphate buffered saline (PBS). Cells were swollen for 10 min in a hypotonic lysis buffer (20-mM HEPES-NaOH, pH 7.9, 10-mM KCl, 1.5-mM MgCl_2_, 0.5-mM dithiothreitol (DTT) and 0.2% NP-40) and collected in a 1.5-ml tube. After centrifugation, nuclear pellets were lysed in chromatin immunoprecipitation (ChIP) lysis buffer (50-mM Tris-HCl, pH 7.9, 1% Triton X-100, 0.1% SDS, 0.1% deoxycholic acid, 10-mM EDTA, 5-mM β-D-glycerophosphate, 1-mM sodium fluoride, 1-mM sodium orthovanadate and 1-mM PMSF), subjected sonication to shear the chromatin to ∼500-bp-long DNA in size, and diluted with four volumes of ChIP dilution buffer (12.5-mM Tris-HCl, pH 7.9, 187.5-mM NaCl and 1% TritonX-100). Clarified lysates by centrifugation were incubated with each antibody or anti-Flag M2 affinity gel (SIGMA) slurry at 4ºC overnight. Antibody-protein–DNA complexes were incubated with Protein A Sepharose 4 Fast Flow (Amersham) and washed with a low salt wash buffer (20-mM Tris-HCl, pH 7.9, 100-mM NaCl, 2-mM EDTA, 0.1% SDS and 1% Triton X-100), with a high salt wash buffer (20-mM Tris-HCl, pH 7.9, 500-mM NaCl, 2-mM EDTA, 0.1% SDS and 1% Triton X-100), with a LiCl buffer (10-mM Tris-HCl, pH 7.9, 250-mM LiCl, 1% NP-40, 1% deoxycholate acid and 1-mM EDTA), and twice with Tris-EDTA (TE), successively. Then, bound proteins were eluted from the beads in an elution buffer (1% SDS and 100-mM NaHCO_3_) by incubation at room temperature for 15 min. Cross-linking was reversed by incubation at 65°C overnight. All samples were treated with 40 μg/ml of Proteinase K (Nacalai Tesque) at 55°C for 2 h, and then DNA was extracted with phenol:chloroform:isoamyl alcohol and precipitated with ethanol. DNA fragments were subjected to qRT-PCR using FastStart SYBR Green Master (Roche) with specific primer sets, 5′-CATGCCTCGGGAAAGGGAAAC-3′ and 5′-GTGACATCTGCCTTACCATGG-3′ for the *ISG15* promoter (−123/+96), 5′-GCAGGAATTCCGCTAGCTTT-3′ and 5′-ATCCTTACCTCATGGTTGCTG-3′ for the *ISG56* promoter (−138/+103), 5′-ACCTCATTGGTCCCTGGCTA-3′ and 5′-AGAAGTGTGGTTTTCTGCGT-3′ for the *IFITM1* promoter (−214/−39), 5′-ACTCAGGTTTGCCCTGAGGGA-3′ and 5′-TGCCTTATGGAGTGCTCCGGTG-3′ for the *IL8* promoter (−124/+12), 5′-GCTCAGGGTTTAGGCTTCTT-3′ and 5′-TATAAACGCTGGCTGGGGAT-3′ for the *IκBα* promoter (−139/−13) and 5′-TCGGTGCGTGCCCAGTTGAACCAG-3′ and 5′-AACAGGAGGAGCAGAGAGCGAAGC-3′ for the *GAPDH* promoter (−212/+18).To measure the amount of TAF-I on the chromatin, HeLa S3 cells transfected with pU6-puro-siTAF-I together with siRNA-resistant Flag-TAF-Iα expression vector were subjected to puromycin selection. In this TAF-I KD/rescure condition, ChIP assays were carried out as described above.

### MNase protection assay

Cells (∼1 × 10^6^) were treated with or without IFN-β (1000 IU/ml) for indicated periods followed by fixation with 1% formaldehyde at room temperature for 15 min. Fixation was quenched by the addition of glycine at the final concentration of 125 mM, and cells were washed twice with ice-cold PBS. The cells were then collected by centrifugation, resuspended in 1.5 ml of Buffer A (300-mM sucrose, 35-mM Tris-HCl, pH 7.9, 15-mM NaCl, 5-mM MgCl_2_, 60-mM KCl, 3-mM CaCl_2_, 0.2% Triton X-100) and incubated at 4ºC for 10 min. The pellet was collected by centrifugation and resuspended in 1.4 ml of MNase Buffer (35-mM Tris-HCl, pH 7.9, 15-mM NaCl, 5-mM MgCl_2_, 60-mM KCl, 3-mM CaCl_2_, 12.5% glycerol), and then chromatin suspension was prepared. The chromatin suspension was divided into two aliquots (300 ul), and 30 units MNase were added to one aliquot. Both aliquots were incubated at room temperature for 5 min with constant rotation, and then EDTA (25 mM at the final concentration) and SDS (1% at the final concentration) were added to stop the reaction. Cross-linking was reversed by incubation at 65°C overnight. All samples were treated with Proteinase K and RNase A to digest proteins and residual RNA, respectively. DNA was extracted with phenol:chloroform:isoamyl alcohol and precipitated with ethanol. DNA fragments were subjected to qRT-PCR using FastStart SYBR Green Master (Roche) with specific primer sets as shown in the ‘Chromatin immunoprecipitation assay’ section of the Materials and Methods section.

### Reporter gene assay

HEK293 cells were transfected with pU6-puro-siTAF-I or pU6-puro-siEGFP together with pISRE-TA-*Luc* (Clontech) containing the ISRE and pSEAP-Control (Clontech) using Gene Juice (Novagen) according to the manufacturer's protocol. At 48 h post transfection, cells were treated with or without IFN-β (1000 IU/ml) for 6 h. Cell lysates were used for assays of the luciferase activity using the luciferase assay system (Promega) and a MiniLumat LB9506 luminometer (Berthhold). To monitor the transfection efficiency, a portion of each cell culture medium was assayed for secreted alkaline phosphate (SEAP) using the SEAP assay kit (Toyobo).

## RESULTS

### TAF-I is involved in ISG transcriptional regulation

**Figure 1.**
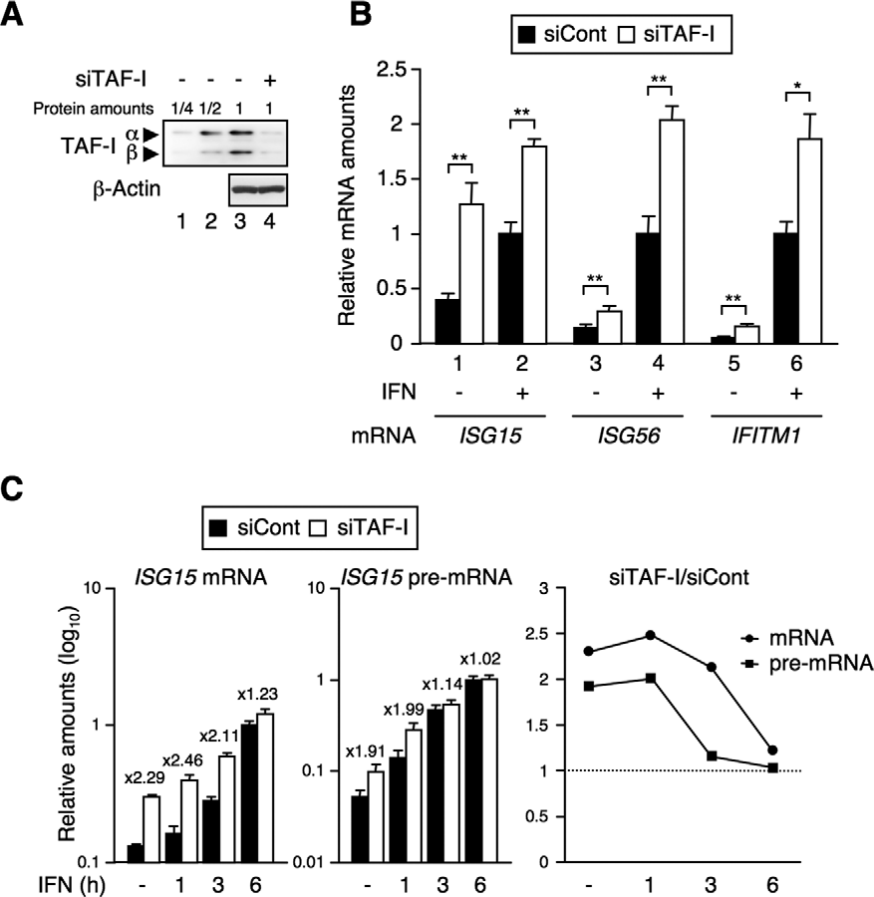
TAF-I negatively regulates ISG transcription. (**A**) Expression level of TAF-I in TAF-I KD cells. HeLa S3 cells were transfected with EGFP siRNA expression vector used as a control (siCont, lanes 1–3) or with TAF-I siRNA expression vector (siTAF-I, lane 4). Cell lysates were subjected to western blot analysis using anti-TAF-I and anti-β-actin antibodies. (**B**) and (**C**) Effects of TAF-I KD in ISG transcription. Total RNA was prepared from siCont- and siTAF-I-transfected cells treated with or without IFN-β for 3 h (B) or for indicated periods (C) and subjected to qRT-PCR using specific primer sets for *ISG* mRNAs, *ISG15* pre-mRNA and *GAPDH* mRNA. The amount of *ISG* mRNA was normalized as a relative amount of *GAPDH* mRNA. The amounts of *ISG15* mRNA and *ISG15* pre-mRNA in TAF-I KD cells relative to those of control cells were shown in the right panel of (C). Error bars represent standard deviation (*n* ≥ 3). *P < 0.01, **P < 0.001 by two-tail-paired Student's *t*-test (*n* = 8).

To study the involvement of TAF-I in ISG transcription, we first examined the effect of TAF-I KD on ISG transcription. TAF-I consists of two subtypes, TAF-Iα and TAF-Iβ. They have common structures except for a 37 amino acid (aa) amino-terminal region of TAF-Iα that differs from a 24 aa amino-terminal region of TAF-Iβ (33). In TAF-I KD cells (see the Materials and Methods section), the levels of the TAF-I proteins were ∼25% of those in control cells (Figure 1A). To examine the effects of the reduction of TAF-I on the transcriptional induction of ISGs, total RNA was isolated from cells and subjected to qRT-PCR with primer sets specific for *ISG* mRNAs (*ISG15*, *ISG56* and *IFITM1*). The *ISG* mRNA levels induced by IFN stimulation were significantly higher in TAF-I KD cells than in control cells (Figure 1B). We also observed the same effect in other TAF-I KD cells transfected with siRNA against TAF-I, targeting a region of TAF-I different from that of the vector expressing siRNA against TAF-I used for the analysis in Figure 1 (Supplementary Figure S1A and B). Note that the increases in *ISG* mRNAs in TAF-I KD cells were observed not only in an IFN-stimulated condition but also in an unstimulated condition. Kinetic analysis showed that the levels of *ISG15* mRNA and *ISG15* pre-mRNA were found to be significantly higher in TAF-I KD cells than those in unstimulated cells at the early stages following IFN induction (Figure 1C). The mRNA levels of the other *ISGs* also showed similar effects in a time-dependent manner (Supplementary Figure S1C). These results indicate that TAF-I negatively regulates ISG transcription and suggest that TAF-I could be involved in the maintenance of the transcriptionally silent state of *ISGs*.

### TAF-I does not affect STAT phosphorylation and ISRE-dependent transcription

**Figure 2.**
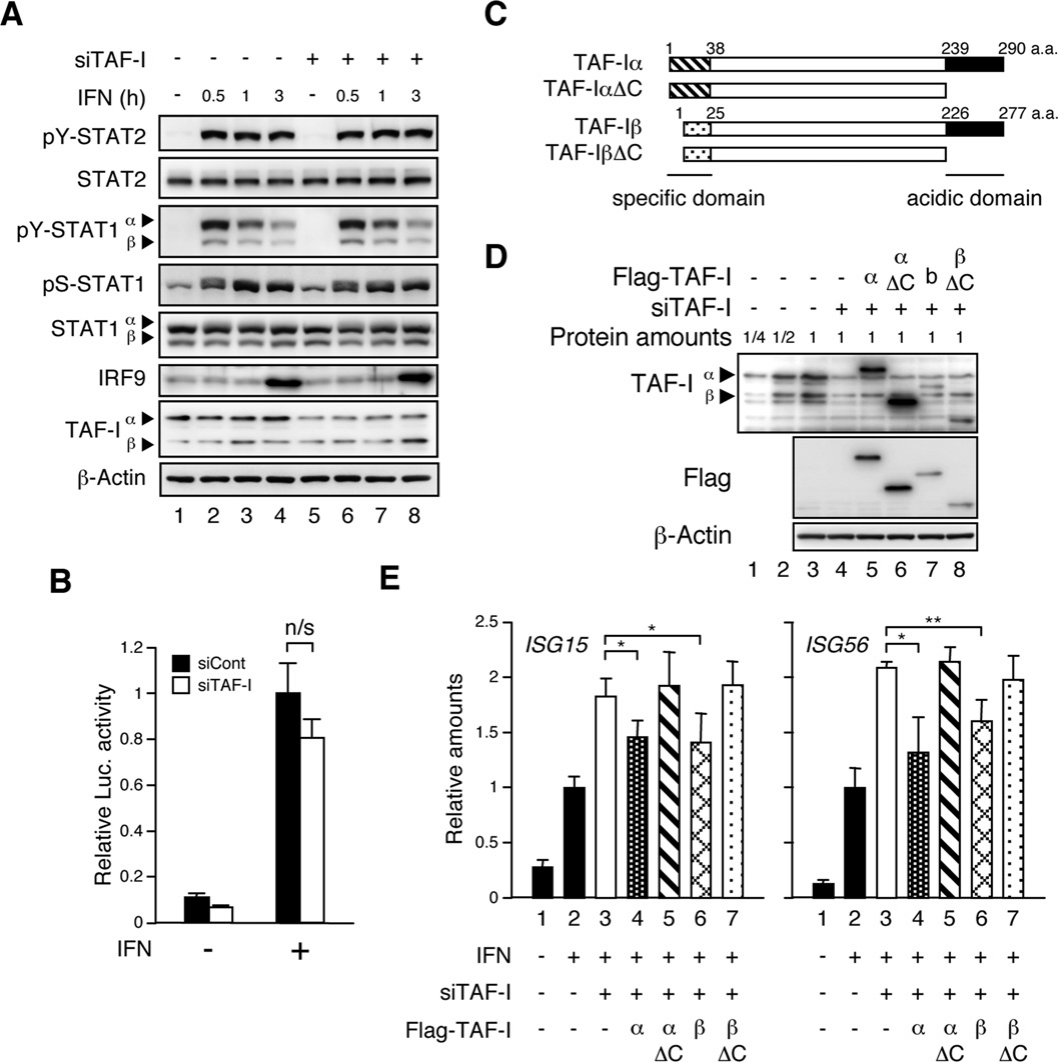
The acidic domain of TAF-I is essential for ISG transcriptional regulation. (**A**) IFN-responsive STAT phosphorylation in TAF-I KD cells. Cell lysates were prepared from siCont- (lanes 1–4) and siTAF-I-transfected (lanes 5–8) cells treated with or without IFN-β for indicated periods, and subjected to western blot analysis using anti-tyrosine phosphorylated (pY)-STAT1, anti-pY-STAT2, anti-serine phosphorylated (pS)-STAT1, anti-STAT1, anti-STAT2, anti-IRF9, anti-TAF-I and anti-β-actin antibodies. (**B**) Dispensability of TAF-I in ISRE-dependent transcription. HEK293 cells were transfected with EGFP siRNA expression vector used as a control (siCont) or with TAF-I siRNA expression vector (siTAF-I) together with pISRE-TA-*Luc* and pSEAP-control, and then cells were treated with or without IFN-β for 6 h followed by the luciferase assay. The luciferase activity was normalized with the SEAP activity and represented as fold-induction relative to that from IFN-treated control cells. Error bars represent standard deviation (*n* ≥ 3). ‘n/s’ indicates ‘not significant’. (**C**) Schematic representation of TAF-I proteins. The N-terminal regions specific for TAF-Iα (aa 1–37) and TAF-Iβ (aa 1–24) and the C-terminal acidic region for TAF-Iα (aa 239–290) and TAF-Iβ (aa 226–227) are indicated. (**D**) Expression level of Flag-TAF-Is in TAF-I KD cells. Cell lysates were prepared from HeLa S3 cells transfected with EGFP siRNA expression vector used as a control (lanes 1–3) or TAF-I siRNA expression vector (siTAF-I, lanes 4–8) together with expression vectors expressing Flag-tagged TAF-Iα (lane 5), Flag-tagged TAF-IαΔC (lane 6), Flag-tagged TAF-Iβ (lane 7), Flag-tagged TAF-IβΔC (lane 8) or empty vector (lane 4), and cell lysates were subjected to western blot analysis using anti-TAF-I, anti-Flag and anti-β-actin antibodies. (**E**) Transcription rescue experiments by Flag-TAF-I. Cells were prepared as shown in (D) and treated with or without IFN-β for 3 h. Total RNA was subjected to qRT-PCR using specific primer sets for *ISG15* mRNA (left), *ISG56* mRNA (right) and *GAPDH* mRNA. The amount of *ISG* mRNA was normalized as a relative amount of *GAPDH* mRNA. Error bars represent standard deviation (*n* ≥ 3). *P < 0.05, **P < 0.01 by two-tail-paired Student's *t*-test (*n* = 6).

To clarify the role of TAF-I in transcriptional regulation, we examined the effect of TAF-I on the IFN signaling pathway leading to transcriptional activation. IFN stimulates the JAK-STAT pathway and induces the phosphorylation of STAT1 and STAT2, leading to the formation of STAT1-STAT2 heterodimers, together with IRF9, forms the ISGF3 complex. The ISGF3 complex binds to ISREs near the *ISG* promoters, facilitates transcription initiation complex formation and thereby promotes the transcription of ISGs (3). The phosphorylation of STAT2 at tyrosine 690 and that of STAT1 at tyrosine 701 are required for STAT1-STAT2 heterodimerization and ISGF3 complex formation, and, additionally, the phosphorylation of STAT1 at serine 727 stimulates activation of ISG transcription (5). To examine whether TAF-I affects the phosphorylation state of STATs, we performed western blot analysis using antibodies specific for each phosphorylated STAT. There were no significant differences in the levels of tyrosine phosphorylated STAT1 and STAT2, serine phosphorylated STAT1 or in the amounts of STAT1, STAT2 and IRF9 between TAF-I KD and control cells (Figure 2A), suggesting that TAF-I does not affect the IFN-induced STAT activating cascade.

To examine whether TAF-I affects the ISRE-dependent transcription directly, we performed reporter gene assays using pISRE-TA-*Luc*, encoding the *luciferase* (*Luc*) gene under the control of the ISRE, which is responsible for Type I IFN stimulation. There was no significant change in luciferase activity between the control and TAF-I KD cells (Figure 2B), indicating that TAF-I does not play a direct role in ISRE-dependent transcription regulated by the ISGF3 complex. This seems to contradict the results presented in Figure 1, where the ISG mRNA levels were increased in TAF-I KD cells. Transcription from transiently transfected genes and stably integrated genes is expressed differently due to nucleosome structure (37–39), the association with histone H1 with the chromatin (40) and the effects of the distal elements on the chromatin (41). Because TAF-I possesses histone chaperone activity (29–31), and it is a major component of the INHAT complex (11), we hypothesized that TAF-I may control ISG transcription through chromatin structure regulation. The C-terminal acidic domain common to TAF-Iα and β is essential for its histone chaperone activity and INHAT activities (11,29–31,33). We constructed expression vectors for TAF-IΔC mutants lacking the acidic domain (Figure 2C) and performed rescue experiments to examine whether the histone chaperone activity and/or INHAT activity are essential for the effects of TAF-I on ISG transcription. The expression of siRNA target sequence-modified Flag-tagged TAF-Is was observed in TAF-I siRNA expression vector-transfected cells (Figure 2D), and the ISG transcription that was enhanced by TAF-I KD was repressed by wild-type TAF-I but not TAF-IΔC (Figure 2E), indicating that the acidic domain of TAF-I, which is essential for the histone chaperone activity, plays a role in the regulation of the ISG transcription.

### The levels of transcription factors and histone H1 on the *ISG* promoters are regulated by TAF-I

**Figure 3.**
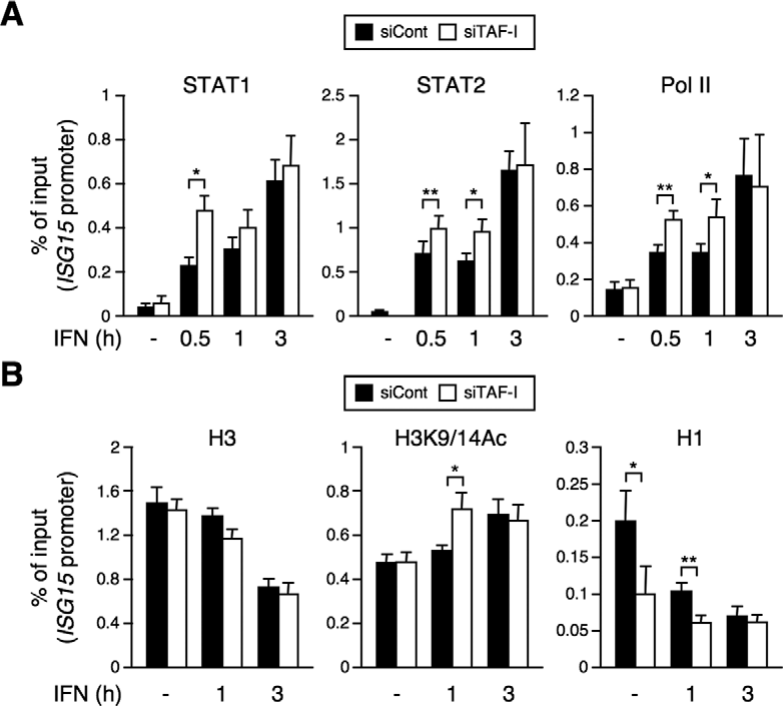
TAF-I regulates the amounts of the transcription factors and histone H1 on *ISG* promoters. (**A**) Promoter binding of transcription factors in TAF-I KD cells. siCont- and siTAF-I-transfected cells were treated with or without IFN-β for indicated periods, and cell lysates were subjected to ChIP assays using antibodies specific for STAT1 (left), STAT2 (middle) and Pol II (right) followed by qRT-PCR using a specific primer set for the *ISG15* promoter. The amount of DNA co-immunoprecipitated with each antibody was shown as % of input. Error bars represent standard deviation (*n* ≥ 3). *P < 0.05, **P < 0.01 by two-tail-paired Student's *t*-test (*n* = 6). (**B**) Decrease of histone H1 levels on the *ISG* promoter in TAF-I KD cells. Cells were prepared as shown in (A) and cell lysates were subjected to ChIP assays using antibodies specific for histone H3 (H3), acetylated histone H3 (H3K9/14Ac) and histone H1.2 (H1) followed by qRT-PCR using specific primer sets for the *ISG15* promoter. The amount of DNA co-immunoprecipitated with each antibody was shown as % of input. Error bar represents standard deviation (*n* ≥ 3). *P < 0.05, **P < 0.01 by two-tail-paired Student's *t*-test (*n* = 6).

To examine whether TAF-I affects transcriptional complex formation on endogenous *ISG* promoters, we performed ChIP assays to measure the amounts of STAT1, STAT2 and Pol II in the TAF-I KD in the presence or absence of IFN. At the early time point, following IFN stimulation, the levels of STAT1, STAT2 and Pol II bound to the *ISG* promoters were elevated in TAF-I KD cells, compared to control cells (Figure 3A and Supplementary Figure S2A). This was in good agreement with the effects seen for TAF-I KD on ISG transcription. These findings suggest that TAF-I affects transcriptional complex formation on the *ISG* promoter.

We predict that above effects were brought about by TAF-I-mediated changes in the chromatin structure. Thus, we performed ChIP assays using antibodies against histone H3 (H3) and acetylated-histone H3 (H3K9/14Ac), the latter of which is known to be associated with actively transcribed genes and the acetylation level of which is regulated by promoter-associated HATs such as p300/CBP and GCN5 (42). qRT-PCR analyses indicated no significant differences in the amounts of histone H3 on *ISG* promoters when treated with or without IFN, between TAF-I KD cells and control cells. The amount of acetylated histone H3 in TAF-I KD cells remained unchanged from that in control cells in the absence of IFN, but upon IFN stimulation, acetylated histone H3 increased in TAF-I KD cells during the early time (Figure 3B and Supplementary Figure S2B). Because STAT interacts with HATs and recruits them to *ISG* promoters in an IFN-dependent manner (6), the increased amount of acetylated-histone H3 in TAF-I KD cells may be due to an increased amount of STAT on the *ISG15* promoter (Figure 3A). Furthermore, ChIP assays using an antibody against linker histone H1.2 (H1), one of the histone H1 subtypes highly expressed in HeLa cells (43) and that is associated with TAF-I *in vivo* (29), demonstrated that the amount of histone H1 on *ISG* promoters in TAF-I KD cells was significantly reduced in the absence of IFN as well as early time point upon IFN stimulation (Figure 3B and Supplementary Figure S2B). These results suggest that TAF-I regulates the amount of histone H1 on the *ISG* promoter but does not act as a histone H3-H4 chaperone or an INHAT in ISG transcription.

### Linker histone H1 is involved in ISG transcriptional regulation

**Figure 4.**
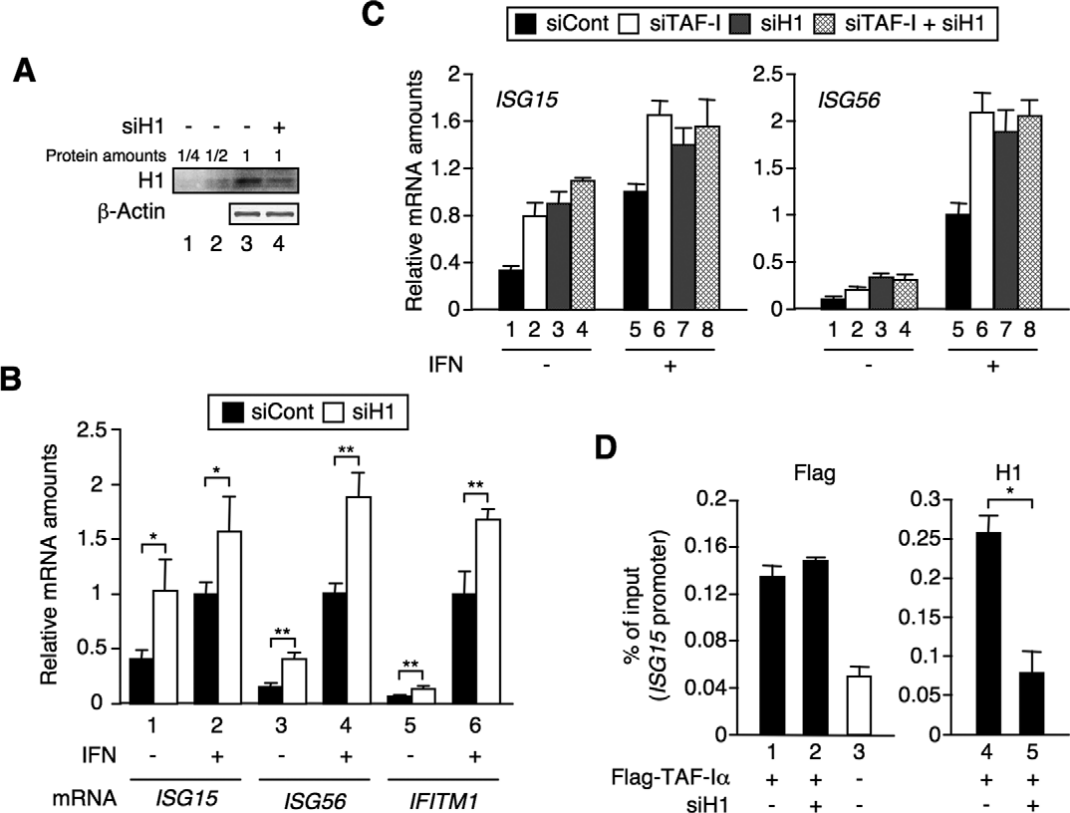
Histone H1 negatively regulates ISG transcription. (**A**) Expression level of histone H1 in histone H1 KD cells. HeLa S3 cells were transfected with EGFP siRNA expression vector used as a control (siCont, lanes 1–3) or histone H1 siRNA expression vector (siH1, lane 4). Cell lysates were subjected to western blot analysis using anti-histone H1.2 (H1) and anti-β-actin antibodies. (**B**) Effects of histone H1 KD on ISG transcription. Total RNA was prepared from siCont- and siH1-transfected cells treated with or without IFN-β for 3 h and subjected to qRT-PCR using specific primer sets for each *ISG* mRNA and *GAPDH* mRNA. The amount of *ISG* mRNA was normalized as relative amounts of *GAPDH* mRNA. Error bars represent standard deviation (*n* ≥ 3). *P < 0.05, **P < 0.01 by two-tail-paired Student's *t*-test (*n* = 4). (**C**) Genetic interaction between TAF-I and histone H1 in the ISG transcription regulation. HeLa S3 cells were transfected with EGFP siRNA expression vector (lanes 1 and 5) and TAF-I siRNA expression vector (lanes 2, 4, 6 and 8) together with or without histone H1 siRNA expression vector (lanes 3, 4, 7 and 8), and then treated with or without IFN-β for 3 h. Total RNA was subjected to qRT-PCR using specific primer sets for *ISG* mRNAs and *GAPDH* mRNA. The amount of *ISG* mRNA was normalized as a relative amount of *GAPDH* mRNA. Error bars represent standard deviation (*n* ≥ 3). (**D**) Binding of TAF-I on *ISG* promoters in histone H1 KD cells. HeLa S3 cells were transfected with TAF-I siRNA expression vector (lanes 1–5) together with empty vector (lane 3) or Flag-tagged TAF-Iα expression vector (lanes 1, 2, 4 and 5), and then transfected with siRNA specific for histone H1.2 (lanes 2 and 5) or negative control siRNA (lanes 1 and 4). Cells were, then, subjected to ChIP assays using the agarose-conjugated antibody against Flag (Flag) and an antibody specific for histone H1.2 (H1) followed by qRT-PCR using specific primer sets for *ISG* promoters. The amount of DNA co-immunoprecipitated with each antibody was shown as % of input. Error bars represent standard deviation (*n* ≥ 3). *P < 0.001 by two-tail-paired Student's *t*-test (*n* = 6).

Next, we addressed the question of whether histone H1 regulates ISG transcription. We prepared histone H1 KD cells using a histone H1 siRNA expression vector. Using this vector, the residual level of histone H1 protein in the histone H1 KD cells was observed to be ∼25% that of the control cells (Figure 4A). To examine the effects on transcriptional induction of ISGs, we measured the levels of *ISG* mRNAs in histone H1 KD cells. The *ISG* mRNA level was increased in histone H1 KD cells compared to that in the control cells, as it was in TAF-I KD cells (Figure 4B). We also observed the same effects in histone H1 KD cells transfected with siRNA against histone H1.2 that targets a region of histone H1.2 different from that of siRNA expression vector used for the analysis in Figure 4 (Supplementary Figure S3). These results indicate that histone H1 acts as a negative regulator of ISG transcription, which correlated well with the decreased amount of histone H1 on the *ISG* promoter in TAF-I KD cells (Figure 3B). Furthermore, we examined the effects of a double KD of TAF-I and histone H1 on ISG transcription (Figure 4C). The *ISG* mRNA level was increased in TAF-I and histone H1 KD cells, but no additive effect was observed in the double KD cells, suggesting that TAF-I and histone H1 may act in the same pathway during the process of ISG transcription. Furthermore, to determine whether the level of histone H1 on the *ISG* promoter affects the chromatin binding of TAF-I, we measured the amount of TAF-I on the *ISG* promoter in histone H1 KD cells (Figure 4D). The amount of histone H1 was significantly decreased on the *ISG* promoter in histone H1 siRNA-transfected cells (Figure 4D, right panel), while the amount of TAF-I was unchanged in histone H1 KD and the control cells (Figure 4D, left panel). These results suggest that histone H1 on *ISG* promoters does not affect chromatin binding of TAF-I.

### TAF-I and histone H1 affect the chromatin structure at the *ISG* promoter

**Figure 5.**
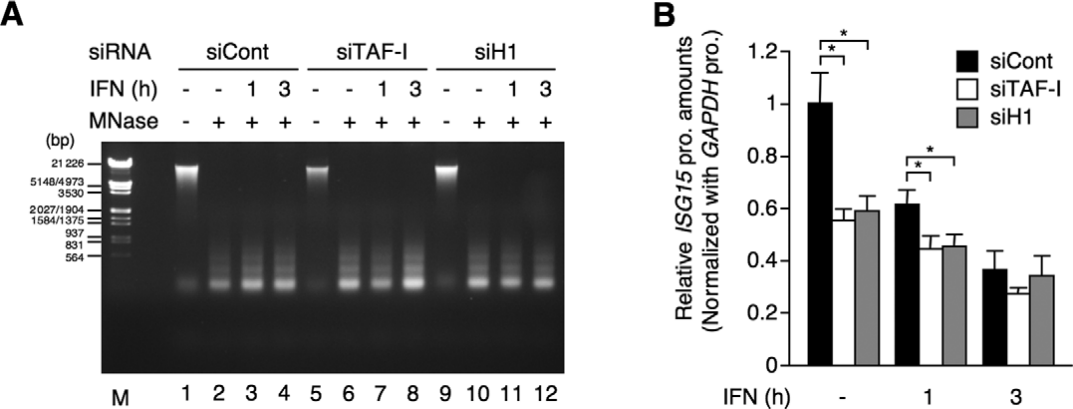
TAF-I and histone H1 regulate the chromatin structure of *ISG* promoters. (**A**) Nucleosome digestion by MNase. HeLa S3 cells were transfected with EGFP siRNA expression vector (siCont, lanes 1–4), TAF-I siRNA expression vector (siTAF-I, lanes 5–8) and histone H1 siRNA expression vector (siH1, lanes 9–12), and then treated with or without IFN-β for indicated periods followed by the fixation with formaldehyde. MNase digestion was carried out as described in the Materials and Methods section, and then genomic DNAs were purified, separated by electrophoresis in 1% agarose gel and visualized by ethidium bromide staining. Lane M shows DNA size markers. (**B**) Increase of the MNase sensitivity of the *ISG15* promoter in TAF-I KD and H1 KD cells. MNase-digested DNA was prepared as shown in (A) and subjected to qRT-PCR using specific primer sets for the *ISG15* and *GAPDH* promoters. The amount of the *ISG15* promoter DNA was normalized by that of the *GAPDH* promoter DNA and is shown as a relative amount to that from IFN-untreated control cells. Error bars represent standard deviation (*n* ≥ 3). *P < 0.0001 by two-tail-paired Student's *t*-test (*n* = 9).

Based on the above results, it is reasonable to postulate that TAF-I regulates the amount of histone H1 on *ISG* promoters and that ISG transcription is negatively controlled by histone H1 in the transcriptionally silent state and during the early stage of ISG transcription induced by IFN stimulation. Histone H1 plays a role in the formation of the chromatin structure at several promoter regions (44–46). To examine if TAF-I and histone H1 are involved in modulating the chromatin architecture near *ISG* promoters, we carried out MNase protection assays, to monitor the compactness of the chromatin in TAF-I KD and histone H1 KD cells (47,48). Overall DNA digestion patterns (nucleosome repeat lengths) seemed to be similar in all cases, suggesting that KD of TAF-I and histone H1.2 or the IFN treatment does not affect the genome-wide nucleosome structure (Figure 5A). Thus, the depletion levels of histone H1.2 in this experimental condition may not be enough to induce the significant changes of the genome-wide nucleosome repeat length as reported in the previous report (49), and it is possible that the positioning of histone H1 via TAF-I is not a genome-wide mechanism, but is instead restricted to the chromatin of certain genes. We then performed qRT-PCR using primers covering *ISG* promoter regions to determine the sensitivity to MNase digestion. The amount of residual genomic DNA from the MNase digestion was significantly reduced in TAF-I and histone H1 KD cells compared to control cells in the transcriptionally silent state and during the early stage of transcription induced by IFN stimulation (Figure 5B and Supplementary Figure S4). These results suggest that TAF-I recruits histone H1 onto *ISG* promoters and hence acts to maintain a relatively close chromatin structure near the *ISG* promoter region to prevent loading of the transcriptional machinery and/or transcription by Pol II.

### TAF-I is associated with *ISG* promoters in the transcriptionally silent state, but dissociated during the early stage of transcription induced by IFN stimulation

**Figure 6.**
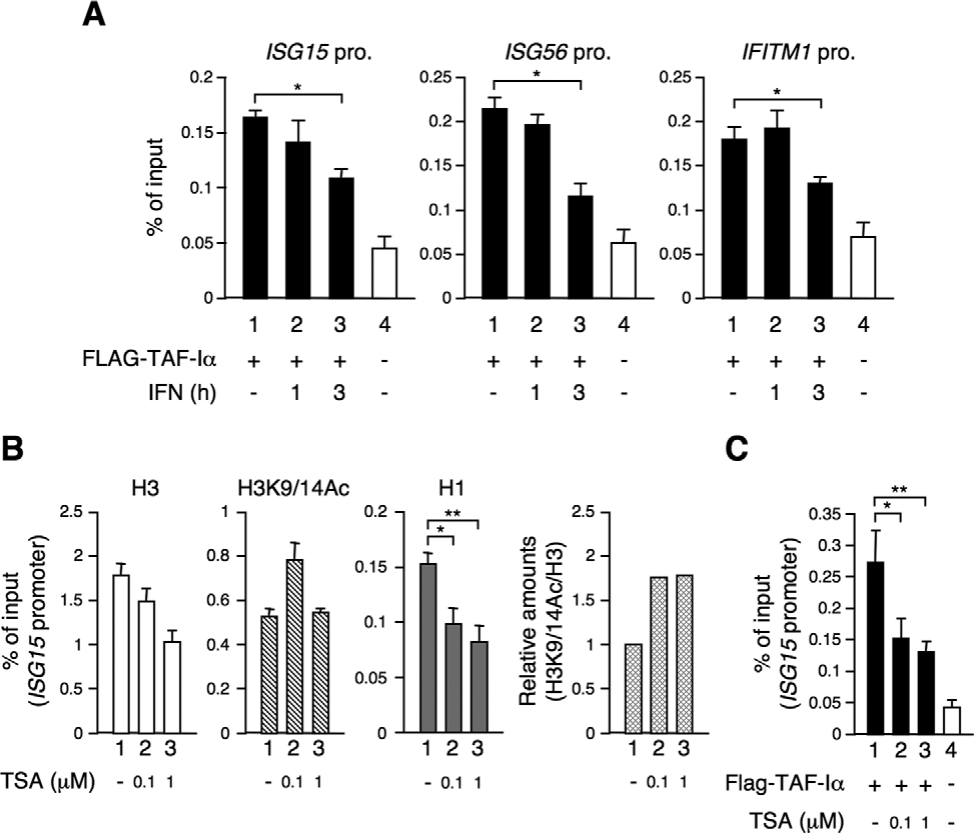
Dissociation of TAF-I from *ISG* promoters. (**A**) Dynamics of TAF-I at *ISG* promoters in an IFN-dependent manner. HeLa S3 cells were transfected with TAF-I siRNA expression vector (lanes 1–4) together with empty vector (lane 4) or Flag-tagged TAF-Iα expression vector (lanes 1–3) and then treated with or without IFN-β for indicated periods. Cells were, then, subjected to ChIP assays using the agarose-conjugated antibody against Flag followed by qRT-PCR using specific primer sets for each *ISG* promoter. The amount of DNA co-immunoprecipitated with each antibody was shown as % of input. Error bars represent standard deviation (*n* ≥ 3). *P < 0.001 by two-tail-paired Student's *t*-test (*n* = 6). (**B**) Effects of TSA treatment on binding of histone H1 to the *ISG15* promoter. HeLa S3 cells were treated without (lane 1) or with 0.1 (lane 2) or 1 μM (lane 3) of TSA for 1 h and then subjected to ChIP assays using antibodies specific for histone H3 (H3), acetylated histone H3 (H3K9/14Ac) and histone H1.2 (H1) followed by qRT-PCR using a specific primer set for the *ISG15* promoter. The amount of DNA co-immunoprecipitated with each antibody was shown as % of input. Error bars represent standard deviation (*n* ≥ 3). *P < 0.001, **P < 0.0001 by two-tail-paired Student's *t*-test (*n* = 6). The relative amount of H3K9/14Ac for H3 is shown in H3K9/14Ac/H3 (fourth panel). (**C**) Effect of TSA treatment on binding of TAF-I to the *ISG15* promoter. Cells were prepared as shown in (A) and treated without (lane 1) or with 0.1 (lane 2) or 1 μM (lane 3) of TSA for 1 h and subjected to ChIP assays using the agarose-conjugated antibody against Flag followed by qRT-PCR using a specific primer set for the *ISG15* promoter. The amount of DNA co-immunoprecipitated with each antibody was shown as % of input. Error bars represent standard deviation (*n* ≥ 3). *P < 0.01, **P < 0.001 by two-tail-paired Student's *t*-test (*n* = 6).

To clarify the dynamics of TAF-I in ISG transcription, we investigated how TAF-I associates with the *ISG* promoter. TAF-I was associated with the *ISG* promoter in the transcriptionally silent state and was gradually released following IFN stimulation (Figure 6A). It is quite possible that the decrease in TAF-I results in a reduction of the amount in histone H1 on *ISG* promoter regions in an IFN-dependent manner (Figure 3B). Previous studies have shown that TAF-I tends to interact with unacetylated and hypoacetylated, but not with hyperacetylated histones (50). We showed that the acetylated histone level is increased at the *ISG* promoter in IFN-treated cells (Figure 3B). Based on these results, we hypothesized that the acetylated histone induced by IFN treatment would restrict binding of TAF-I to the *ISG* promoter. To test this hypothesis, we performed ChIP assays using chromatin prepared from HeLa S3 cells treated with TSA, an HDAC inhibitor, to maintain high levels of histone acetylation at the *ISG* promoter (Figure 6B and Supplementary Figure S5A). Indeed, TSA treatment promoted histone H3 acetylation. Furthermore, the amount of histone H1 on the *ISG15* promoter was significantly decreased in TSA-treated cells compared to mock-treated cells. Under these conditions, we also measured the amount of TAF-I bound to the *ISG* promoter, using cells prepared by the same method as for Figure 6A followed by TSA treatment. The level of TAF-I bound to the *ISG* promoter was significantly decreased in the TSA-treated cells (Figure 6C and Supplementary Figure S5B). A possible interpretation of these results is that the acetylated histone induced by IFN treatment restricts TAF-I from binding to the *ISG* promoter, which in turn prevents histone H1 recruitment onto the *ISG* promoter during the late stages of the ISG transcription.

## DISCUSSION

In this study, we clarified the functional interplay between TAF-I and histone H1 in the silencing of ISG transcription and the maintenance of the transcriptionally silent state of *ISG*. We found that the ISG transcription level was increased in TAF-I KD cells compared with control cells in the transcriptionally silent state and during the early stage of transcription induced by IFN stimulation. The ISG transcription was aberrantly enhanced by TAF-I KD even in the absence of IFN, suggesting that TAF-I might have a role in the silencing mechanism of ISG transcription in the unstimulated state. In TAF-I KD cells, the histone H1 level at the *ISG* promoter was significantly reduced, whereas the amounts of the transcriptional complexes and acetylated-histone H3 on the *ISG* promoter were increased. In addition, the histone H1 level at the *ISG* promoter did not affect the chromatin binding by TAF-I. The ISG transcription level was enhanced in histone H1 KD cells as well as TAF-I KD cells compared with control cells. The C-terminal acidic domain of TAF-I, which is responsible for its histone chaperone activity, was required for the repression of ISG transcription. Double KD experiments suggested that the target of TAF-I is histone H1 in the ISG transcriptional regulation process. In TAF-I KD and histone H1 KD cells, the chromatin structure of the *ISG* promoter was shown to be more relaxed compared to that of control cells. Histone H1 plays a major role in the formation of higher order chromatin structures (17–19) and affects the binding of the transcription factors to DNA, the activity of the ATP-dependent chromatin remodeling factors and HAT-dependent acetylation of histone (44–46,51–54). These results strongly suggest that TAF-I regulates the chromatin structure of *ISG* promoters through histone H1 chaperone activity and that the chromatin binding of histone H1, which is regulated by TAF-I, inhibits the binding of transcriptional complexes to the *ISG* promoter.

TAF-I was associated with the *ISG* promoter in the transcriptionally silent state and was released after IFN stimulation (Figure 6A), consistent with its role in ISG transcriptional regulation. The amount of acetylated histone on the *ISG* promoter was increased in a time-dependent manner after IFN treatment and correlated well with the increased transcription factors bounding to the promoter (Figure 3). This might be due to the recruitment of GCN5 through STAT2, followed by histone acetylation on the *ISG* promoter in an IFN-dependent manner (6). The acetylated histone level on the *ISG* promoter induced by IFN stimulation, along with the TAF-I dissociation, results in a decrease in the amount of histone H1 on the *ISG* promoter (Figures 3B and 6A). This is in good agreement with the previous report that TAF-I binds to unacetylated, hypoacetylated and repressively modified histones, but not to hyperacetylated histones (50). These results are consistent with the results shown in Figure 6B and C, where TSA treatment showed restrict binding of TAF-I and histone H1 to the *ISG* promoter.

We propose that TAF-I recruits histone H1 onto the *ISG* promoter and hence maintains a relatively close chromatin structure at the *ISG* promoter to prevent aberrant ISG transcription independent of IFN stimulation. STATs bring HATs onto the *ISG* promoter in an IFN-dependent manner, leading to an increase in the level of acetylated histone, which in turn causes dissociation of TAF-I from the *ISG* promoter. Consequently, the amount of histone H1 on the *ISG* promoter is reduced, followed by the formation of an open chromatin structure that allows optimal binding of transcriptional complexes and subsequent transcription by Pol II.

Note that the inhibition of HDAC activity leads to the global impairment of the ISG transcription, and this may be due to the impairment of Pol II recruitment onto the *ISG* promoter (8–10). These studies imply that the basal level of histone acetylation on the *ISG* promoter in the unstimulated condition maintains the promoter in the transcriptionally silent state but does so as to enable the rapid induction of transcription by IFN stimulation. We showed that histone H1 dynamics are co-ordinated with TAF-I dynamics at *ISG* promoters in an IFN- and a TSA-dependent manner. Collectively, it is possible that TAF-I may continuously regulate the placement and positioning of histone H1 of certain genes, and it may not only act on gene silencing maintenance but also in permitting responses to transcription inducer.

In this report, we focused on histone H1.2 as a target of TAF-I in ISG transcriptional regulation, and we observed significant effects of histone H1.2 KD in each experiment, implying that at least histone H1.2 plays a role in ISG transcriptional regulation. Indeed, genome-wide studies suggested that there are differential features for histone H1 variants in their distribution on the genome and in the role for the genes expression (49,55). On the other hand, the expression levels and the distribution of each histone H1 variant are strongly dependent on cell types, such as cultured cell lines, tissues and developmental stages (43,49,55,56). In addition, TAF-I interacts with not only histone H1.2 but also other histone H1 variants *in vivo* (29). Therefore, we could not exclude the possibility that histone H1 variants other than H1.2 also have a role in ISG transcription through the histone H1 chaperone activity of TAF-I.

Recent genome-wide studies suggested that histone H1 is present at proximal and distal gene promoters and is depleted from proximal promoters including TSS in transcription activation-dependent manner (55,57–59). Note that histone H1 depletion was facilitated at proximal promoters of inducible genes under a stimulating condition (55), and this is in good agreement with our results. The other important question is whether the regulatory mechanism by TAF-I and histone H1 is specific for *ISG* locus. To address if TAF-I and histone H1 affect other inducible systems, we examined the effect of TAF-I and histone H1 KD on the transcription of *IL8* and *IκBα*, which are dependent on NF-κB (Supplementary Figure S6). In the TAF-I and histone H1 KD cells, the level of *IL8* mRNA was increased in the TAF-I and histone H1 KD cells in a TNF-α-unstimulated condition (Supplementary Figure S6A). The amount of histone H1 on the *IL8* promoter in TAF-I KD cells was reduced similar to the *ISG* promoters (Supplementary Figure S6B and Figure 3B), suggesting that TAF-I and histone H1 may regulate *IL8* transcription through a regulatory mechanism similar to that for *ISG*. In contrast, the level of *IκBα* mRNA was not increased in TAF-I nor histone H1 KD. Thus, the regulatory mechanism that depends on TAF-I and histone H1 is not restricted to ISG transcription but is not a general mechanism that controls all inducible promoters. There are no consensus sequences in the proximal promoter regions between the *ISG* and *IL8* genes, so that a specific transcription factor(s), which recognizes the consensus sequence, may not play a role in the recruitment of TAF-I on the chromatin. Recent genome-wide studies have revealed that the promoter/enhancer regions exhibit a characteristic chromatin structure consisting of specific modification, nucleosome positioning and specific histone variants including histone H1s (1,55,60). In addition, distal enhancer elements affect gene transcription through enhancer–promoter communication by forming chromatin loop structures (41,61,62). The distal enhancer elements may also play roles in recruiting negative regulators, such as TAF-I, to the proximal promoter for the maintenance of gene silencing.

## SUPPLEMENTARY DATA

Supplementary Data are available at NAR Online.

SUPPLEMENTARY DATA
