# Supplementary material for: Silencing of IFN-stimulated gene transcription is regulated by histone H1 and its chaperone TAF-I
Source: Nucleic Acids Res. 2014 Jun 14;42(12):7642–53. doi: 10.1093/nar/gku485 (PMC4081089; doi:10.1093/nar/gku485)
Supplement: SUPPLEMENTARY DATA [file supp_42_12_7642__index.html]

Silencing of IFN-stimulated gene transcription is regulated by histone H1 and its chaperone TAF-I — Silencing of IFN-stimulated gene transcription is regulated by histone H1 and its chaperone TAF-I — Silencing of IFN-stimulated gene transcription is regulated by histone H1 and its chaperone TAF-I — SUPPLEMENTARY DATA 

# Silencing of IFN-stimulated gene transcription is regulated by histone H1 and its chaperone TAF-I

## SUPPLEMENTARY DATA

**Files in this Data Supplement:**

- SUPPLEMENTARY DATA
