# Supplementary material for: Silencing of IFN-stimulated gene transcription is regulated by histone H1 and its chaperone TAF-I
Source: Nucleic Acids Res. 2014 Jun 14;42(12):7642–53. doi: 10.1093/nar/gku485 (PMC4081089; doi:10.1093/nar/gku485)

Silencing of IFN-stimulated gene transcription is regulated by histone H1 and its chaperone TAF-I

Shinichi Kadota and Kyosuke Nagata

Supplementary Figure Legends

Supplementary Figure S1. TAF-I negatively regulates ISG transcription. (A) Expression level of TAF-I in TAF-I KD cells. HeLa S3 cells were transfected with siRNA specific for TAF-I (siTAF-I, lane 4) or negative control siRNA (siNC, lanes 1-3). Cell lysates were subjected to western blot analysis using anti-TAF-I and anti-β-actin antibodies. (B) Effects of TAF-I KD in ISG transcription. Total RNA was prepared from siNC- and siTAF-I-transfected cells treated with or without IFN-β for 3 hours, and subjected to qRT-PCR using specific primer sets for each *ISG* mRNAs and *GAPDH* mRNA. The amount of *ISG* mRNA was normalized as a relative amount of *GAPDH* mRNA. Error bars represents standard deviation (n ≥ 3). $*P < 0.05$, $**P < 0.001$ by two-tail paired Student's *t*-test (n = 8). (C) Effect of TAF-I KD in time-dependent manner. HeLa S3 cells were transfected with EGFP siRNA expressing vector used as a control (siCont) or with TAF-I siRNA expressing vector (siTAF-I). Total RNA was prepared from siCont- and siTAF-I-transfected cells treated with or without IFN-β for indicated periods, and subjected to qRT-PCR using specific primer sets for *ISG56* mRNAs, *IFITM1* mRNA, and *GAPDH* mRNA. The amount of *ISG* mRNA was normalized as a relative amount of *GAPDH* mRNA. Error bars represents standard deviation (n ≥ 3). The amount of *ISG* mRNA in TAF-I KD cells relative to that of siCont cells was shown in right panel of (C).

Supplementary Figure S2. TAF-I regulates the amounts of transcription factors and histone H1 on *ISG* promoters. (A) Promoter binding of the transcriptional factors in TAF-I KD cells. siCont- and siTAF-I-transfected cells were treated with or without IFN-β for indicated periods, and cell lysates were subjected to ChIP assays using antibodies specific for STAT1 (left), STAT2 (middle), and Pol II (right) followed by qRT-PCR using specific primer sets for the *ISG56* and the *IFITM1* promoters. The amount of DNA co-immunoprecipitated with each antibody was shown as % of input. Error bars represents standard deviation ($n \geq 3$). $^*P < 0.01$, $^{**}P < 0.001$ by two-tail paired Student's *t*-test ($n = 6$). (B) Decrease of histone H1 levels on *ISG* promoters in TAF-I KD cells. Cells were prepared as shown in (A), and cell lysates were subjected to ChIP assays using antibodies specific for histone H3 (H3), acetylated histone H3 (H3K9/14Ac), and histone H1.2 (H1) followed by qRT-PCR using specific primer sets for the *ISG56* and the *IFITM1* promoters. The amount of DNA co-immunoprecipitated with each antibody was shown as % of input. Error bars represents standard deviation ($n \geq 3$). $^*P < 0.05$, $^{**}P < 0.01$, $^{***}P < 0.001$ by two-tail paired Student's *t*-test ($n = 6$).

Supplementary Figure S3. Histone H1.2 negatively regulates ISG transcription. (A) Expression level of histone H1.2 in histone H1 KD cells. HeLa S3 cells were transfected with siRNA specific for histone H1.2 (siH1.2, lane 4) or negative control siRNA (siNC, lanes 1-3). Cell lysates were subjected to western blot analysis using anti-histone H1.2 and anti-β-actin antibodies. (B) Effects of histone H1.2 KD on ISG transcription. Total RNA was prepared from siNC- and siH1.2-transfected cells treated with or without IFN-β for 3 hours and subjected

to qRT-PCR using specific primer sets for each *ISG* mRNA and *GAPDH* mRNA. The amount of *ISG* mRNA was normalized as a relative amount of *GAPDH* mRNA. Error bars represents standard deviation (n ≥ 3). *P < 0.05, **P < 0.01, **P < 0.001 by two-tail paired Student's *t*-test (n = 6).

Supplementary Figure S4. Involvement of TAF-I and histone H1 in the chromatin structure of *ISG* promoter regions. The MNase protection assay for *ISG* promoter regions in TAF-I KD and H1 KD cells was carried out. MNase-digested DNA was prepared as shown in Figure 5A and subjected to qRT-PCR using specific primer sets for *ISG56*, *IFITM1*, and *GAPDH* promoter regions. The amounts of *ISG* promoter region DNAs were normalized by the amount of the *GAPDH* promoter region DNA, and shown as a relative amount to that from IFN-untreated siCont cells. Error bars represents standard deviation (n ≥ 3). *P < 0.001, *P < 0.0001 by two-tail paired Student's *t*-test (n = 9).

Supplementary Figure S5. Dissociation of TAF-I from *ISG* promoters. (A) Effects of TSA treatment on binding of histone H1 to *ISG* promoters. HeLa S3 cells were treated without (lane 1) or with 0.1 (lane 2) or 1 μM (lane 3) of TSA for 1 hour and subjected to ChIP assays using antibodies specific for histone H3 (H3), acetylated histone H3 (H3K9/14Ac), and histone H1 (H1) followed by qRT-PCR using specific primer sets for *ISG56* and *IFITM1* promoters. The amount of DNA co-immunoprecipitated with each antibody was shown as % of input. Error bars represents standard deviation (n ≥ 3). *P < 0.001, **P < 0.0001 by two-tail paired Student's *t*-test (n = 6). The amount of H3K9/14Ac relative to that of H3 is shown in

H3K9/14Ac/H3 (4th panel). (B) Effect of TSA treatment on binding of TAF-I to *ISG* promoters. HeLa S3 cells were transfected with TAF-I siRNA expressing vectors (lanes 1-4) together with empty vector (lane 4) or Flag-tagged TAF-Iα expressing vector (lanes 1-3), and were treated without (lane 1) or with 0.1 (lane 2) or 1 μM (lane 3) of TSA for 1 hour and subjected to ChIP assays using the agarose-conjugated antibody against Flag followed by qRT-PCR using specific primer sets for the *ISG56* and the *IFITM1* promoters. The amount of DNA co-immunoprecipitated with antibody was shown as % of input. Error bars represents standard deviation (n ≥ 3). $*P < 0.001$ by two-tail paired Student's *t*-test (n = 6).

Supplementary Figure S6. TAF-I and histone H1 negatively regulate *IL8* gene transcription. **(A)** Effects of TAF-I KD and histone H1 KD in *IL8* transcription. Total RNA was prepared from siCont- (lanes 1 and 4), siTAF-I- (lanes 2 and 5) and siH1- (lanes 3 and 6) transfected cells and subjected to qRT-PCR using specific primer sets for *IL8* mRNAs, *IκBα* mRNA, and *GAPDH* mRNA. The amount of each mRNA was normalized as a relative amount of *GAPDH* mRNA. Error bars represents standard deviation (n ≥ 3). $*P < 0.01$, $**P < 0.0001$ by two-tail paired Student's *t*-test (n = 4). (B) Decrease of the histone H1 level on the *IL8* promoter in TAF-I KD cells. Cell lysates prepared from siCont- and siTAF-I-transfected cells were subjected to ChIP assays using an antibody specific for histone H1.2 (H1) followed by qRT-PCR using specific primer sets for the *IL8* and *IκBα* promoters. The amount of DNA co-immunoprecipitated with each antibody was shown as % of input. Error bars represents standard deviation (n ≥ 3). $*P < 0.0001$ by two-tail paired Student's *t*-test (n = 4).

Kadota, S. et al. Supplementary Figure S1

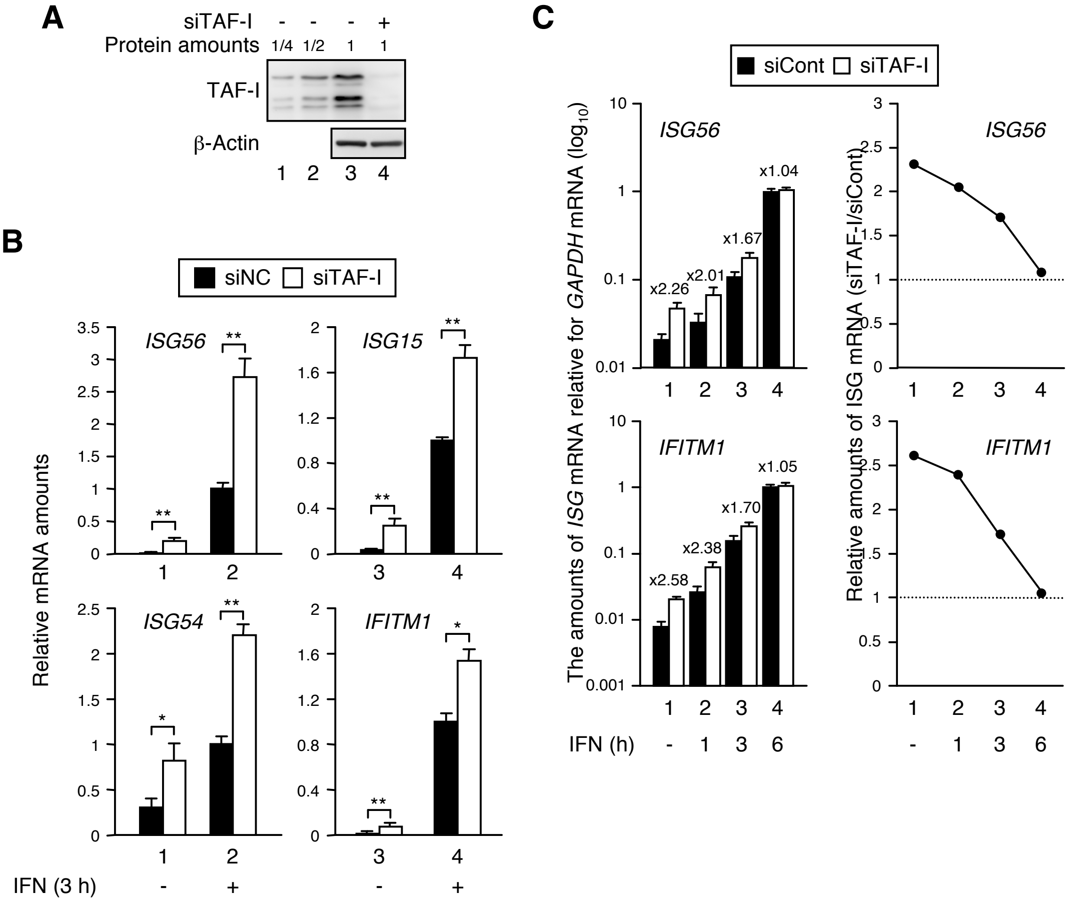

Kadota, S. et al. Supplementary Figure S2

A

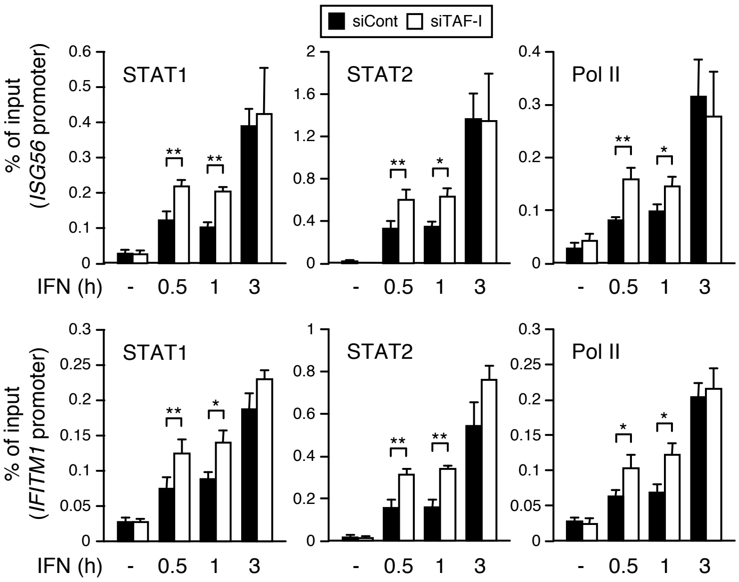

B

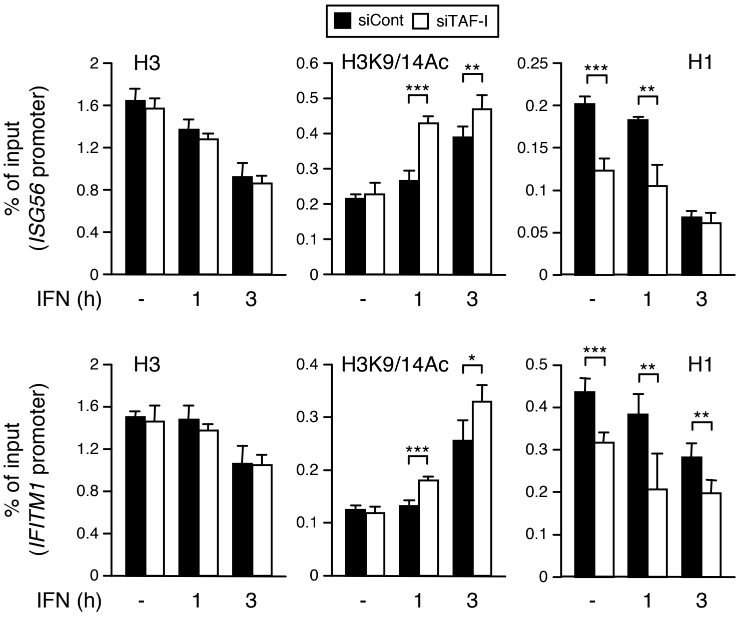

Kadota, S. et al. Supplementary Figure S3

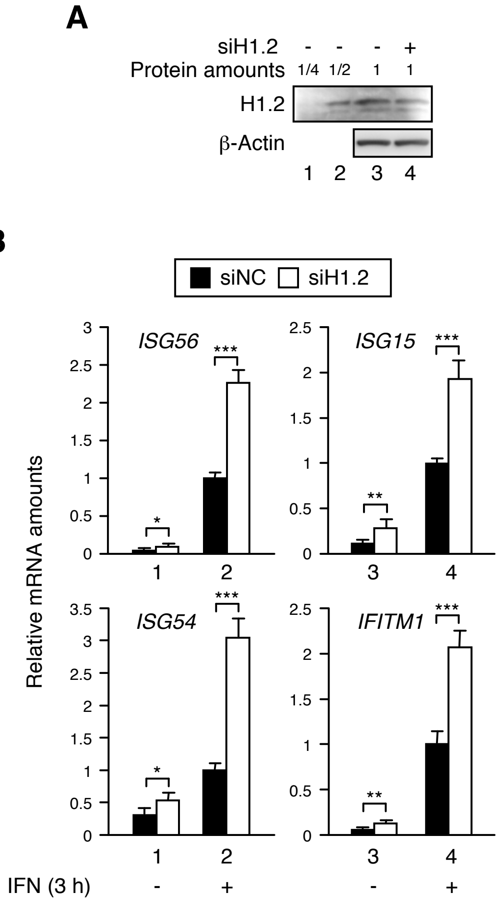

Kadota, S. et al. Supplementary Figure S4

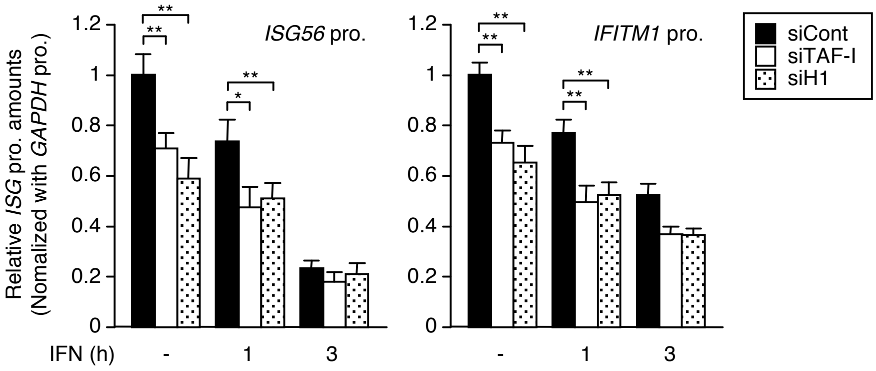

Kadota, S. et al. Supplementary Figure S5

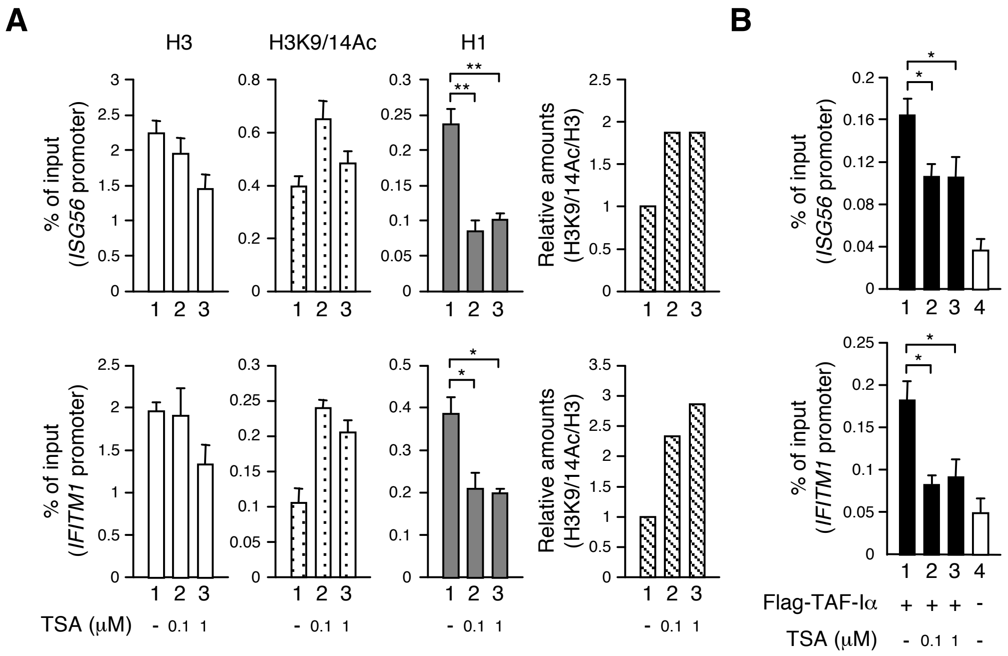

Kadota, S. et al. Supplementary Figure S6

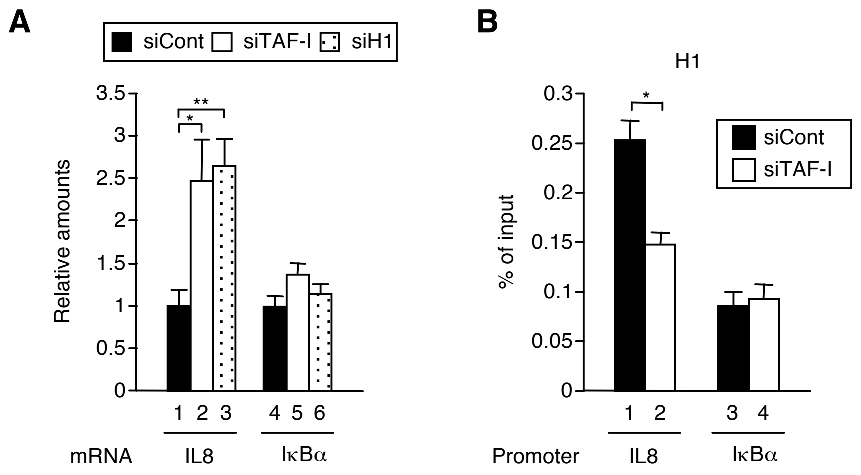

Supplement: SUPPLEMENTARY DATA [file supp_gku485_nar-00438-v-2014-File008.pdf]
